# Acute Myeloid Leukemia Presenting As Spontaneous Splenic Rupture: A Case Report

**DOI:** 10.7759/cureus.105539

**Published:** 2026-03-20

**Authors:** Abdellah Seghiri, Ikram Sadki, Zahida Aqodad, Samia Sabri, Houda Bachir, Siham Hamaz, Habiba Alaoui, Khalid Serraj

**Affiliations:** 1 Department of Internal Medicine, Immunohematology and Cellular Therapy Laboratory, Mohammed VI University Hospital, Medical School of Oujda, Mohammed First University of Oujda, Oujda, MAR

**Keywords:** acute myeloid leukemia ( aml), emergency abdominal surgery, hemoperitoneum, spontaneous splenic rupture, total splenectomy

## Abstract

Spontaneous splenic rupture (SSR) is a rare but potentially life-threatening condition that may occur in the setting of underlying splenic pathology, including hematologic malignancies such as acute myeloid leukemia (AML). We report the case of a 28-year-old man who presented with sudden-onset severe abdominal pain. Imaging revealed massive hemoperitoneum secondary to SSR. Laboratory investigations demonstrated severe anemia, thrombocytopenia, leukocytosis, and circulating blasts on peripheral smear, with normal coagulation parameters. The patient underwent urgent splenectomy, and histopathological examination showed extensive infiltration by myeloperoxidase-positive blasts. Bone marrow aspiration and immunophenotyping confirmed AML with a complex karyotype and adverse prognostic features. Despite initiation of induction chemotherapy, the clinical course was complicated by septic shock leading to death. This case highlights SSR as a rare initial manifestation of AML and emphasizes the importance of early recognition, prompt imaging, and multidisciplinary management to improve clinical outcomes.

## Introduction

Although rare, spontaneous splenic rupture (SSR) is a life-threatening medicosurgical emergency requiring prompt recognition and management. It accounts for less than 1% of all splenic ruptures. Diagnosis remains challenging, particularly in the absence of any history of trauma [1,2].

SSR most commonly occurs in a diseased spleen affected by underlying conditions such as infections, inflammatory disorders, neoplasms, or hematologic malignancies [3]. Hematologic diseases represent a recognized cause of atraumatic splenic rupture, accounting for a significant proportion of reported cases. The proposed mechanisms include leukemic infiltration of the splenic parenchyma and capsule, vascular congestion, splenic infarction with subsequent subcapsular hematoma formation, and coagulation abnormalities [4,5].

In some patients, SSR may represent the first manifestation of an undiagnosed hematologic malignancy, leading to rapid hemodynamic collapse and carrying a high risk of mortality [4]. We report the case of a young patient in whom SSR revealed acute myeloid leukemia (AML) with a complex karyotype, highlighting the diagnostic challenge, the rarity of this presentation, and its poor prognostic implications.

## Case presentation

A 28-year-old male patient, followed since childhood for cerebellar ataxia secondary to vitamin E deficiency, was admitted to the emergency department with sudden-onset diffuse abdominal pain. There were no associated digestive symptoms, in particular no bowel disturbances or gastrointestinal bleeding (no hematemesis, melena, or rectorrhagia), and no extradigestive manifestations.

On physical examination, the patient was conscious but appeared pale and tachycardic (115 beats/minute), hypotensive (90/50 mmHg), and afebrile, and had an oxygen saturation of 98%. Abdominal examination revealed a distended, tense abdomen with marked tenderness predominating in the left upper quadrant. Hernial orifices were free. No peripheral lymphadenopathy or clinically palpable hepatosplenomegaly was detected. Cardiovascular and respiratory examinations were unremarkable.

**Figure 1:**
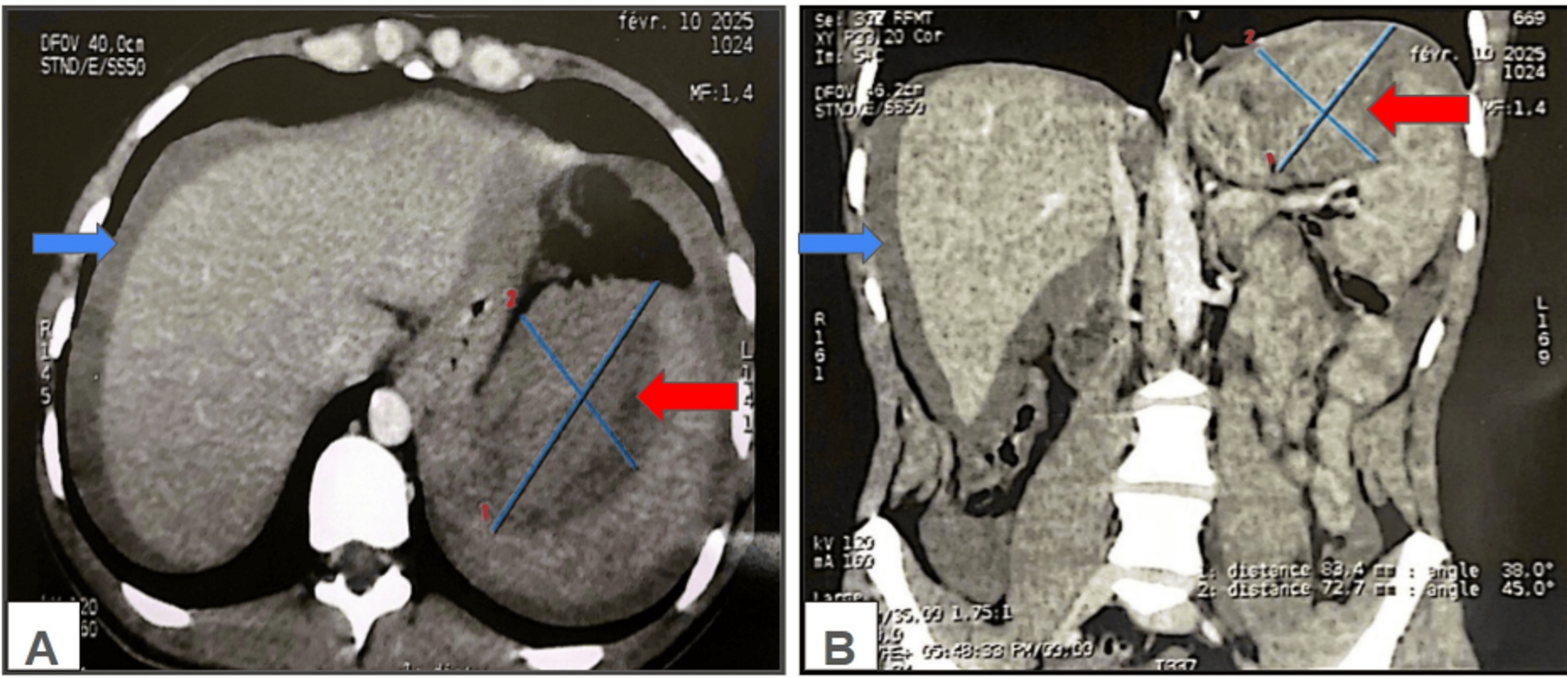
Abdominal computed tomography scan showing splenic capsular rupture with an associated perisplenic hemorrhagic collection. (A) Axial view. (B) Coronal view. Red arrows represent the splenic capsular rupture, and blue arrows represent the areas of hemoperitoneum

Abdominal ultrasound revealed a large splenic collection suggestive of a hematoma. An urgent abdominopelvic computed tomography (CT) scan confirmed the presence of massive hemoperitoneum secondary to capsular splenic rupture. The spleen was of normal size, and no intra-abdominal or pelvic lymphadenopathy was identified (Figure 1).

Initial laboratory tests showed a normochromic, normocytic, nonregenerative anemia (hemoglobin 6.3 g/dL), thrombocytopenia (82 × 10⁹/L), and marked hyperleukocytosis (32 × 10⁹/L). The differential count revealed a predominance of neutrophils (25 × 10⁹/L) and lymphocytes (2.5 × 10⁹/L). Peripheral blood smear examination demonstrated 32% circulating blasts. C-reactive protein was elevated at 162 mg/L, with no obvious infectious focus. Lactate dehydrogenase was 560 IU/L, uric acid 80 mg/L, potassium 4.1 mmol/L, creatinine 6.8 mg/L, and the coagulation profile was within normal limits (Table 1).

**Table 1:** Summary of initial laboratory findings with corresponding reference ranges

Parameter	Result	Reference range
Hemoglobin	6.3 g/dL	12-16 g/dL (female)/13-17 g/dL (male)
Mean corpuscular volume	88 fL	80-100 fL
Reticulocyte count	0.08 × 10⁶/µL	0.02-0.1 × 10⁶/µL
Platelet count	82 × 10⁹/L	150-400 × 10⁹/L
Total leukocyte count	32 × 10⁹/L	4-10 × 10⁹/L
Neutrophils	25 × 10⁹/L	1.5-7.5 × 10⁹/L
Lymphocytes	2.5 × 10⁹/L	1.0-4.0 × 10⁹/L
Circulating blasts	32% - myeloid-appearing blasts with Auer rods	0%
C-reactive protein	162 mg/L	<5 mg/L
Lactate dehydrogenase	560 IU/L	140-280 IU/L
Uric acid	80 mg/L	30-70 mg/L
Potassium	4.1 mmol/L	3.5-5.0 mmol/L
Creatinine	6.8 mg/L	6-11 mg/L

**Figure 2:**
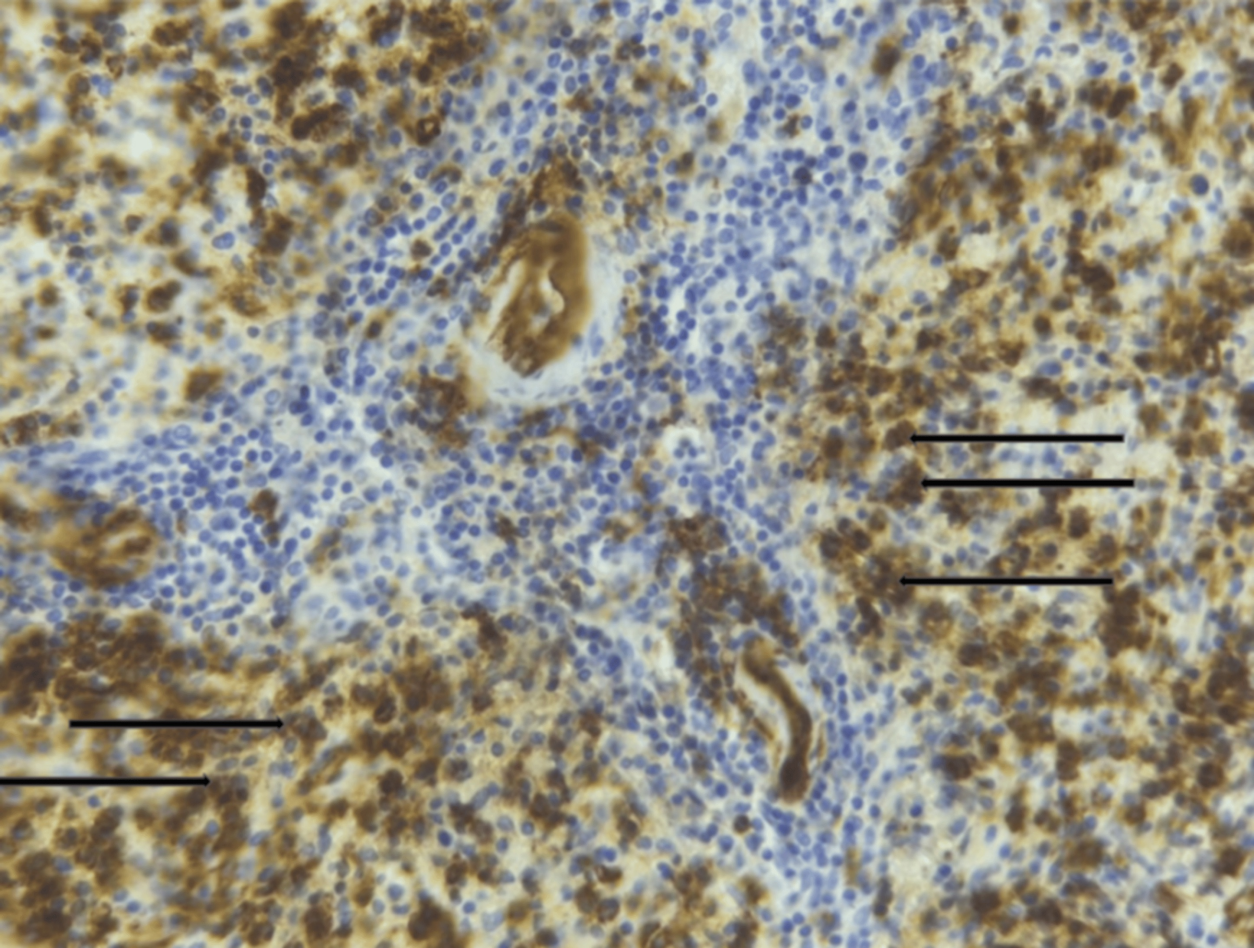
Immunohistochemical staining showing CD34 positivity in splenic blast cells (original magnification ×40). Black arrows represent CD34-positive blast cells

**Figure 3:**
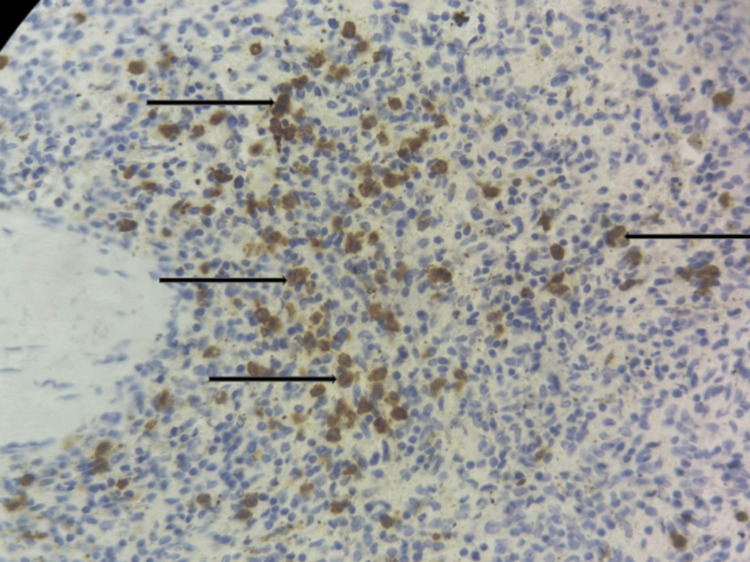
Immunohistochemical staining demonstrating MPO expression in infiltrating splenic blast cells (original magnification ×40). Black arrows represent MPO-positive blast cells MPO: myeloperoxidase

After hemodynamic stabilization and transfusion of two units of packed red blood cells, an emergency splenectomy was performed. Histopathological examination of the splenic specimen revealed globally preserved splenic architecture. The white pulp was composed of lymphoid aggregates surrounding arterioles, within which large blast-like cells were observed. The red pulp showed extensive hemorrhagic changes with marked vascular congestion. Immunohistochemical analysis demonstrated that the blast cells were positive for CD34 (Figure 2) and myeloperoxidase (MPO) (Figure 3), consistent with splenic infiltration by AML.

**Figure 4:**
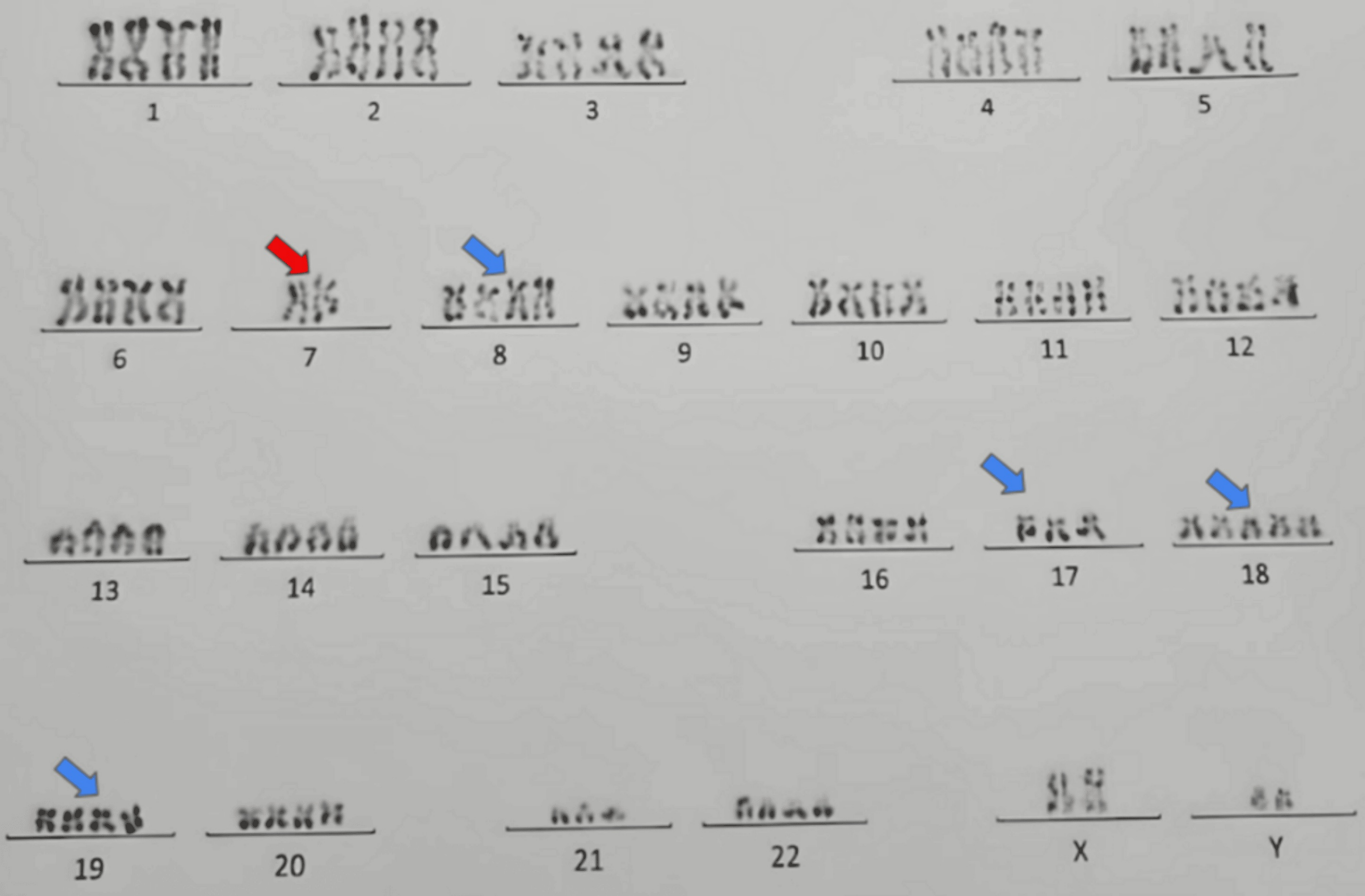
Karyotype showing hyperdiploid clone (90, XXYY, -7, -7) consistent with the duplication of the initial clone and double loss of chromosome 7. Colored arrows indicate chromosomal abnormalities, red arrows mark the double loss of chromosome 7, and blue arrows highlight the duplicated chromosomal set

Bone marrow aspiration revealed massive marrow infiltration with approximately 85% myeloblasts. Flow cytometry confirmed a myeloid immunophenotype (CD13⁺, CD33⁺, MPO⁺). Cytogenetic analysis demonstrated a complex karyotype, with an initial hypodiploid clone (45 chromosomes) harboring monosomy 7, and a hyperdiploid subclone (90 chromosomes) corresponding to duplication of the initial clone with double loss of chromosome 7. This profile classified the patient in the adverse-risk group according to the 2022 European LeukemiaNet (ELN) classification (Figure 4).

Induction chemotherapy was initiated with the “7+3” regimen (continuous infusion cytarabine at 200 mg/m² for seven days combined with daunorubicin at 60 mg/m² for three days). The clinical course was unfavorable, complicated by severe neutropenia and septic shock without an identified focus, leading to the patient’s death despite intensive supportive care.

## Discussion

SSR is a rare complication of AML and may rarely present as the initial manifestation of the disease [5,6]. The pathophysiology of SSR in AML is multifactorial, involving massive leukemic infiltration of the spleen (particularly the capsule), splenic infarction with subsequent subcapsular hematoma formation, and coagulation abnormalities [6,7]. The precise incidence of SSR in AML remains unknown; only a limited number of cases describing SSR as the first manifestation of AML have been reported in the literature [6]. In the present case, rupture occurred during the prediagnostic phase, representing a severe initial presentation.

In a review of 613 cases of nontraumatic splenic rupture, Aubrey-Bassler and Sowers identified infectious, hematologic, neoplastic, and drug-related causes as the most frequent etiologies (Table 2) [3]. Hematologic malignancies accounted for 15% of SSR cases. Within this group, non-Hodgkin lymphomas were the most frequent cause (30%), particularly aggressive and primary splenic forms, followed by acute leukemias (15%) and chronic myeloproliferative disorders, including primary myelofibrosis (10%-15%) [3,6].

**Table 2:** Etiological distribution of nontraumatic splenic rupture (n = 613) Source: Adapted from [3], published under the terms of the Creative Commons Attribution License (http://creativecommons.org/licenses/by/2.0)

Etiology	Percentage (%)
Procedure-related	18.3
Infectious	23.3
Hematologic	13.7
Nonhematologic neoplasms	7.8
Drug-related	7.7
Pregnancy-related	6.2
Other	23

Abdominal ultrasound is the first-line diagnostic modality for SSR, while CT offers higher sensitivity and specificity (≥95%) and allows detailed assessment of lesion severity to guide management [6,8]. Conservative management may be considered in selected patients with intact splenic parenchyma and stable laboratory parameters under close monitoring. Splenectomy is indicated for patients with hemoperitoneum or refractory hypovolemic shock [6,9].

In addition, splenic infiltration by leukemic blasts may represent a form of extramedullary disease in AML. Extramedullary involvement has been associated with aggressive disease biology and poorer outcomes in several studies [10]. In the present case, histopathological examination demonstrated extensive leukemic infiltration of the spleen, which may have contributed to capsular fragility and subsequent rupture.

In this case, the prognosis was poor due to severe initial presentation, hemodynamic instability, high blast count, and complex karyotype. Cytogenetic abnormalities such as monosomy 7 are associated with adverse-risk AML according to the ELN classification and reflect aggressive disease biology and unfavorable clinical outcomes.

## Conclusions

SSR, although rare, may represent a life-threatening initial manifestation of AML. Clinicians should consider an underlying hematologic malignancy in patients presenting with acute abdomen and atraumatic splenic rupture. Early recognition, prompt imaging, and timely surgical intervention are essential to improve survival. Comprehensive hematologic, immunophenotypic, and cytogenetic evaluation remains crucial for diagnosis and risk stratification, as such presentations may reflect aggressive disease with adverse prognosis.
